# In vitro evaluation of the activity of an essential oil from Pistacia vera L. variety Bronte hull against Candida sp.

**DOI:** 10.1186/s12906-018-2425-0

**Published:** 2019-01-07

**Authors:** Manuela D’Arrigo, Carlo Bisignano, Pierangela Irrera, Antonella Smeriglio, Roberto Zagami, Domenico Trombetta, Orazio Romeo, Giuseppina Mandalari

**Affiliations:** 1 Department of Chemical, Biological, Pharmaceutical and Environmental Science, University of Messina, Via SS. Annunziata, 98168 Messina, Italy; 2 Department of Biomedical, Dental, Morphological and Functional Images Sciences, University of Messina, Via C. Valeria, 98125 Messina, Italy; 3 Fondazione Imbesi, Messina, Italy; 4 IRCCS Centro, Neurolesi Bonino-Pulejo, S.S. 113 Via Palermo C.da Casazza, 98123 Messina, Italy

**Keywords:** Pistachio, Essential oil, *Candida* sp., Antifungal

## Abstract

**Background:**

*Candida* sp. represent the most common cause of fungal infections worldwide. In the present work, we have evaluated the activity of an essential oil extracted from pistachio hulls against a number of standard and clinical strains of *Candida* sp.

**Methods:**

*C. albicans* ATCC 64550, *C. parapsilosis* ATCC 22019, 4 clinical strains of *C. albicans*, 3 clinical strains of *C. parapsilosis* and 3 clinical strains of *C. glabrata* were used. All clinical isolates were identified by species-specific PCR-based methods. Susceptibility studies were performed using pistachio hull essential oil alone or in combination with antifungal compounds. The interactions between pistachio hull essential oil and selected antifungal compounds were also evaluated using the checkerboard method and the mechanisms of interaction investigated by droplet size distribution.

**Results:**

Pistachio hull essential oil was fungicidal at the concentrations between 2.50 and 5.0 mg/ml. D-limonene and 3-Carene were the components with major activity. An antagonistic effect was observed with all combinations tested.

**Conclusion:**

The antifungal activity of pistachio hull essential oil could be used to help control resistance in *Candida* species. More studies need to be performed to elucidate the mechanisms responsible for the activity of pistachio hull essential oil.

## Background

Over the last few years, incidence of *Candida spp*. infections have significantly increased, with higher mortality rates and hospital acquired infections [1]. In immunocompetent subjects, *Candida spp*. are generally responsible for mucosal infections, including thrush and vaginitis, which can lead to invasive candidiasis in immunocompromised patients, with emerging species becoming multi-drug resistant [2]. Due to the increased resistance to antifungal compounds, more effort is focused on natural drugs, to use alone or in combination with existing antimicrobials [3]. Essential oils (EOs) are a complex mixture of hydrocarbons and oxygenated hydrocarbons which have recently gained interest due to their antimicrobial potential against human pathogens and their protective role regarding cardiovascular and neurological diseases, diabetes and cancer [4].

Gucwa et al. [5] have recently reported the activity of EOs from *Thymus vulgaris*, *Citrus limonum*, *Pelargonium graveolens*, *Cinnamomum cassia*, *Ocimum basilicum* and *Eugenia caryophyllus* against 183 clinical isolates of *C. albicans* and 76 isolates of *C. glabrata*. The chemical composition and antifungal effects of the *Eugenia uniflora* EO were recently investigated against *C. albicans*, *C. krusei* and *C. tropicalis* standard strains [6].

We have previously characterised the nutraceutical, antioxidant and cytoprotective activity of pistachio (*Pistacia vera* L., variety Bronte) hulls extracts: they are rich in phenols, including flavonols, phenolic acid, and flavan-3-ols, and anthocyanins, such as cyanidyn-3-*O*-galactoside [7, 8]. Furthermore, the antimicrobial activity of the EO from pistachio hulls against Gram-positive and Gram-negative strains, both American Type Culture Collection (ATCC) and clinical isolates, was investigated [9].

In the present study, the EO extracted from pistachio hulls was tested against standard and clinical strains of *C. albicans*, *C. glabrata* and *C. parapsilopsis*, both alone and in combination with antifungal drugs. The molecular identification of the clinical strains is also reported.

## Methods

### Plant material

The hulls of ripe pistachio nuts (*Pistacia vera* L., Bronte variety) were collected in end-August 2017 by a local farmer in Bronte (Catania, Italy) and identified by Prof. Laura Cornara (Botanist at the Dept. DISTAV of the University of Genoa, Italy). A voucher specimen has been deposited in the ChiBioFarAm Department, University of Messina (Messina, Italy).

### Essential oil isolation and characterization

The pistachio hull essential oil was isolated by hydrodistillation using a Clevenger apparatus and characterized by GC-FID and GC-MS analysis according to Smeriglio et al. [9].

### Origin and identification of the Candida strains

All clinical *Candida* isolates tested in this study were recovered at the IRCCS Centro Neurolesi “Bonino-Pulejo” hospital, Messina, Italy, during a surveillance program funded by the Italian Ministry of Health for the prevention and control of healthcare*-*associated fungal infections (project code: GR-2011-02347606). These isolates were obtained from blood samples of patients with acquired brain injuries. Initially, all strains were presumptively identified using *Candida* medium (Becton Dickinson, Italy) and Vitek 2 yeast identification system (bioMérieux, Italy) following manufacturer’s recommendations. The identity of the yeast isolates was subsequently confirmed by using simple and rapid species-specific PCR-based methods according to previous studies [10–12]. Briefly, total genomic DNA was extracted from *Candida* cells using the glass-beads disruption method followed by conventional phenol*/*chloroform*/isoamyl* alcohol purification [13]. I*n vitro* amplifications (total volume 50 μl) were carried out separately for each strain using the Dream Taq Green PCR Master Mix (Thermo Fischer scientific, Milan, Italy), a ready-to-use solution containing all reagents required for PCR to which were only added the genomic DNA template (0.5 μg) and the specific primers (0.5 μM each), depending on the assay type (Table 1). The amplicons were analyzed by 1.5% agarose gel electrophoresis for determining the expected DNA fragment sizes for *C. albicans*, *C. parapsilosis* and *C. glabrata* (Table 1) by excluding the probable cryptic presence of phylogenetically closely related species.

**Table 1:** PCR primers used for molecular identification of *Candida* species tested in this study

Molecular method	Species identified	Primer name	Sequence (5′ → 3′)	Amplicon size	Reference
Singleplex PCR	*C. albicans*	CR-f	GCTACCACTTCAGAATCATCATC	~ 960 bp	Romeo and Criseo, 2008
		CR-r	GCACCTTCAGTCGTAGAGACG		
Multiplex PCR	*C. glabrata*	UNI-5,8S	ACCAGAGGGCGCAATGTG	~ 397 bp	Romeo et al., 2009
		GLA-f	CGGTTGGTGGGTGTTCTGC		
		NIV-f	AGGGAGGAGTTTGTATCTTTCAAC		
		BRA-f	GGGACGGTAAGTCTCCCG		
Multiplex PCR	*C. parapsilosis*	mCPF	TTTGCTTTGGTAGGCCTTCTA	~ 171 bp	Asadzadeh et al., 2015
		mCOF	TAAGTCAACTGATTAACTAAT		
		mCMF	AACTGCAATCCTTTTCTTTCTA		
		mLEF	TACAGAATTTTGAGAATTGTG		
		mCPCR	AATATCTGCAATTCATATTACT		

### Microbial strains and culture conditions

The following strains were used for the antifungal testing: *C. albicans* ATCC 64550, *C. parapsilosis* ATCC 22019, 4 clinical strains of *C. albicans* (12, 13, 16, 17), 3 clinical strains of *C. parapsilosis* (26, 30, 34), 3 clinical strains of *C. glabrata* (9, 25, 33). Strains were grown in RPMI 1640 (Sigma, Italy) at 30 °C for 24 h. For minimal fungicidal determination and killing curves, Sabouraud Dextrose Agar (Oxoid) was used.

### Susceptibility studies

For the susceptibility studies, the EO from pistachio hull was dissolved in DMSO at the concentration of 10 mg/ml. The minimum inhibitory concentration (MIC) and the minimum fungicidal concentration (MFC) of pistachio hull EO and the antifungal compounds voriconazole, fluconazole and caspofungin (Sigma Aldrich, Italy) against the strains reported above were determined following the CLSI guidelines (M27-A3 2008, [14]). Serial dilutions were performed in RPMI 1640 at concentrations between 16 and 0.0156 μg ml^− 1^ (voriconazole), 64 and 0.0625 μg ml^− 1^ (fluconazole), 2 and 0.00195 μg ml^− 1^ (caspofungin) and 10 and 0.0049% mg/ml (pistachio hull essential oil). MFC (minimal fungicidal concentration) for the pistachio hull EO was determined transferring each clear sample (20 μL) on agar plate incubated at 30 °C for 48 h. The MFC was defined as the lowest extract concentration that killed 99.9% of the final inocula after 24-48 h incubation.

In the combination assays, the ‘checkerboard’ procedure was followed [15] in order to test the efficacy of the combination EO/antifungal compounds against all tested strains. This method allows varying the concentrations of each antimicrobial along the different axes, thus ensuring that each well contained a different combination [16]. MIC data for pistachio hull EO and each antifungal compound were converted into fractional inhibitory concentration (FIC), defined as the ratio of the concentration of the antimicrobial in an inhibitory concentration with a second compound to the concentration of the antimicrobial by itself.

FICI = MIC of A with B/MIC of A.

In order to identify the active antifungal components of the pistachio hull EO, MICs and MFCs were also determined with α-pinene, α-terpineol, camphene, D-limonene and 3-carene as well as the mix of these compounds at the concentrations found in the EO against *C. albicans* strain 16, *C. glabrata* strain 9 and *C. parapsilosis* strain 26.

All experiments were performed in triplicate on three independent days.

### Time kill curves

Tubes containing each antifungal compound at concentrations corresponding to 1, 1/2 and 1/4 x MIC were inoculated with a suspension of *C. glabrata* strain 9, *C. albicans* strain 16 and *C. parapsilosis* strain 26, yielding a final fungal density of 5 × 10^5^ CFU/ml and then incubated at 30 °C in a shaking incubator. A growth control was also performed. Samples for viable counting were withdrawn at 0, 1, 2, 4, 8 and 24 h and, if necessary, diluted in fresh medium. At least four dilutions of each sample were spread on agar plates (Sabouraud Dextrose Agar), incubated at 30 °C and counted after 48 h.

### Droplet size and size distribution

Size distribution and average diameter of emulsions’ droplets were determined by dynamic light scattering on a Malvern 4700 submicron analyzer (Malvern Instruments Inc., Worcestershire, U.K.). Distilled water was used as dispersant to avoid effects of multiple scattering, dispersion, and interactions between droplets. The cumulative mean diameter (z-average) and polydispersity index (PdI) were used to describe droplet average size and size distribution, respectively.

## Results

### Antifungal activity of pistachio hull EO

MICs and MFCs values for pistachio hull EO and three antifungal compounds were determined (Table 2). Results of negative controls using DMSO as a solvent at the concentration of 1% (*v*/v) indicated the complete absence of inhibition of all the strains tested (data not shown). Pistachio hull EO was active against all strains tested, *C. parapsilosis* strains being the most sensitive (complete inhibition achieved with a concentration of 1.25–2.50 mg/ml), followed by *C. glabrata* strains (complete inhibition achieved with a concentration of 1.25–5.0 mg/ml) and *C. albicans* strains (complete inhibition achieved with a concentration of 5.0 mg/ml). The effect of pistachio hull EO was fungicidal against all tested strains. Interestingly, all clinical strains of *C. albicans* and1 strain of *C. glabrata* (strain 33) were resistant to both voriconazole and fluconazole.

**Table 2:** MICs and MFCs of Pistachio Essential Oil (expressed as mg/ml) and antifungal compounds (expressed as µg/ml) against *Candida sp*.

STRAIN	VORICONAZOLE		FLUCONAZOLE		CASPOFUNGIN		Pistachio Essential Oil	
	MIC	MFC	MIC	MFC	MIC	MFC	MIC	MFC
*Candida glabrata* strain *9*	0.015–0.031	1	8–8	8	0.015–0.031	1	2.5–5.0	5.0
*Candida glabrata* strain *25*	0.015–0.031	0.031	0.25–0.5	0.5	0.125–0.25	1	5.0	5.0
*Candida glabrata* strain *33*	> 16	/	> 64	/	0.125–0.25	1	1.25–2.50	5.0
*Candida parapsilosis* strain *26*	0.015–0.031	0.031	0.5–1	1	0.5–0.25	0.5	1.25–2.50	2.50
*Candida parapsilosis* strain *30*	0.015–0.031	0.031	0.5–1	1	0.125–0.25	0.5	1.25–2.50	2.50
*Candida parapsilosis* strain *34*	0.125–0.25	0.25	8–4	8	0.25–0.5	0.5	1.25–2.50	2.50
*Candida albicans* strain *12*	> 16	/	> 64	/	0.0075–0.015	0.015	5.0	5.0
*Candida albicans* strain *13*	> 16	/	> 64	/	≤0.0019	/	5.0	5.0
*Candida albicans* strain *16*	> 16	/	> 64	/	0.015	0.015	5.0	5.0
*Candida albicans* strain *17*	> 16	/	> 64	/	≤0.0019	/	5.0	5.0
*Candida albicans ATCC 64550*	1	1	32	32	0.0625	0.0625	2.50–5.0	5.0
*Candida parapsilosis ATCC 22019*	0.062	0.062	4	8	1–2	1	1.25–2.50	2.50

*MIC* Minimum Inhibitory Concentration, *MFC* Minimum Fungicidal Concentration

In the combination assays, the FIC index calculated for pistachio hull EO and each antifungal compound was > 4 against all tested strains. Although the interpretation of the FIC indices depends on which definition is used, here we have interpreted the index as synergistic if the FIC index is ≤0.5, additive or indifferent if > 0.5 but ≤4 and antagonistic if > 4 [16, 17].

Table 3 reports the MIC data for the pure compounds present in pistachio hull EO and the mix of the most representative compounds in the proportion found in the oil. Amongst the pure molecules, 3-carene was the most effective against the three representative tested strains, followed by D-limonene. No activity was detected with α-pinene, α-terpineol and camphene. Interestingly, the mix of compounds was effective against all tested strains, indicating a possible synergistic interaction amongst the individual compounds. As shown in Table 2, *C. albicans* strain 16 was the most resistant, followed by *C. glabrata* strain 9 and *C. parapsilosis* strain 26.

**Table 3:** MICs of pistachio hull EO pure compounds and their mix *C. albicans* strain 16, *C. glabrata* strain 9 and *C. parapsilosis* strain 26

Strain	α-pinene	α-terpineol	Camphene	D-limonene	3-Carene	Mix
*C. albicans* strain 16	> 1000	> 1000	> 1000	125–250	62.50–125	250
*C. glabrata* strain 9	> 1000	> 1000	> 1000	62.50	62.50–125	125–250
*C. parapsilosis* strain 26	> 1000	> 1000	> 1000	31.25–62.50	15.65–31.25	62.5

Values are expressed as μg/ml and represent the mean of three determinations. Standard errors between these three determinations are negligible

### Fungal killing

**Fig. 1:**
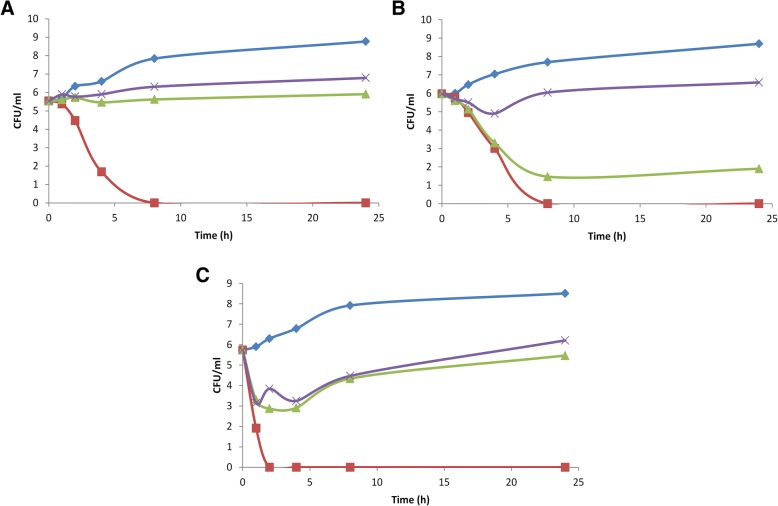
Killing curves for pistachio hull EO against *C. albicans* strain 16 (**a**), *C. glabrata* strain 9 (**b**) and *C. parapsilosis* strain 26 (**c**). Control, ♦; 1/4xMIC, ×; 1/2xMIC, ▲; 1xMIC, ■. Mean of three experiments. MIC = minimum inhibitory concentration

Concentration-dependent killing was observed with pistachio hull EO against all tested strains (Fig. 1 a-c). A good fungicidal effect was achieved after 2 h exposure against *C. parapsilosis* strain 26 at the concentration of 1 x MIC, whereas the same effect was achieved against *C. albicans* strain 16 and *C. glabrata* strain 9 after 8 h exposure. Concentrations of ½ MIC and ¼ MIC exerted a fungistatic effect against all tested strains. As reported in Tables 1 and 2, *C. albicans* strain 16 was the most resistant.

### Light scattering

Table 4 summarizes the effect of the essential oil (EO) on the droplet size and emulsion polydispersity (PdI) in bacteria culture medium, taking into account both the hydrodynamic then the distribution width of this complex system. Both parameters are calculated according to the International Standard on dynamic light scattering, ISO 22412 [18]. The partition coefficient (logP) of the three antifungals under study was calculated by miLogP2.2 software [19].

**Table 4:** Effect of the Pistachio Essential oil on droplet size (Z-Average Diameter) and emulsion polydispersity (Polydispersity index) in yeast culture medium

Preparation	Z-Average Diameter (nm)	Polydispersity index	LogP
RPMI+DMSO	881.4 ± 1.8	0.836 ± 0.011	–
Fluconazole	1570.0 ± 2.1	1.0 ± 0.012	−0.12
Voriconazole	2316.0 ± 2.3	1.0 ± 0.011	1.49
Caspofungin	531.3 ± 1.6	0.679 ± 0.010	−4.59
EO	2151.0 ± 2.2	1.0 ± 0.012	–
EO + Fluconazole	847.5 ± 2.3	0.688 ± 0.008	–
EO + Voriconazole	343.7 ± 1.5	0.426 ± 0.007	–
EO + Caspofungin	690.1 ± 2.1	0.538 ± 0.004	–

Theoretical logP value of the three antifungals are reported. Each measurement represents mean ± standard deviation (*n* = 3)

The droplet size distribution of the different emulsions revealed the lack of a droplet population below 100 nm, while a droplet population with a diameter above than 300 nm was detected.

Pistachio hull EO, fluconazole and voriconazole showed an average diameter greater than 1500 nm and a PdI equal to 1.0, whereas caspofungin showed an average diameter around 500 nm and a PdI of 0.679.

## Discussion

The present study has demonstrated that an essential oil extracted from pistachio hulls was effective against clinical strains of *Candida*. The dramatic rise in antimicrobial resistance is now considered a major health treat, with an estimated 700,000 people dying every year from antibiotic, antiviral, antifungal and antimalarial resistance infections [20]. Therefore, natural products have become an important source for drug development. Geraniol has been tested against *C. albicans* species and its mechanism of action has been elucidated [21]. Ebani et al. [22] have recently demonstrated the activity of five essential oils (star anise, basil, oregano, clary sage and thymus) against multidrug-resistant strains of *Escherichia coli*, *Enterococcus* spp., *C. albicans* and *C. famata* responsible for urinary tract infections. The pistachio hull essential oil tested in the present work was effective against *Candida sp*., whereas polyphenol-rich extracts from pistachios (natural raw shelled and roasted salted pistachios) and white grape juice used in our previous investigations were not effective against yeasts [23, 24]. Amongst the major components identified in the pistachio hull EO, D-limonene and 3-carene were effective against *C. albicans* strain 16, *C. glabrata* strain 9 and *C. parapsilosis* strain 26 (Table 3). This is in agreement with the demonstrated antifungal activity against *C. albicans* of the *Chamaecyparis nootkatensis* essential oil [25].

It is known that plant natural products mostly exert their antifungal effects by membrane-active mechanism and synergistic effects can be found between different classes of plant products as well as between natural products and azoles [26]. Bioactive compounds present in natural products, such as EOs, may interact to produce synergistic, additive or antagonistic effects. Synergistic interactions could result in increased efficacy, or reduce effective doses, therefore reducing the likelihood of adverse effects [27]. We have previously shown that pairwise combinations of polyphenols present in almonds (protocatechuic acid, naringenin and epicatechin) showed both synergistic and indifferent interactions against *Salmonella enterica* and *Staphylococcus aureus* [28].

**Fig. 2:**
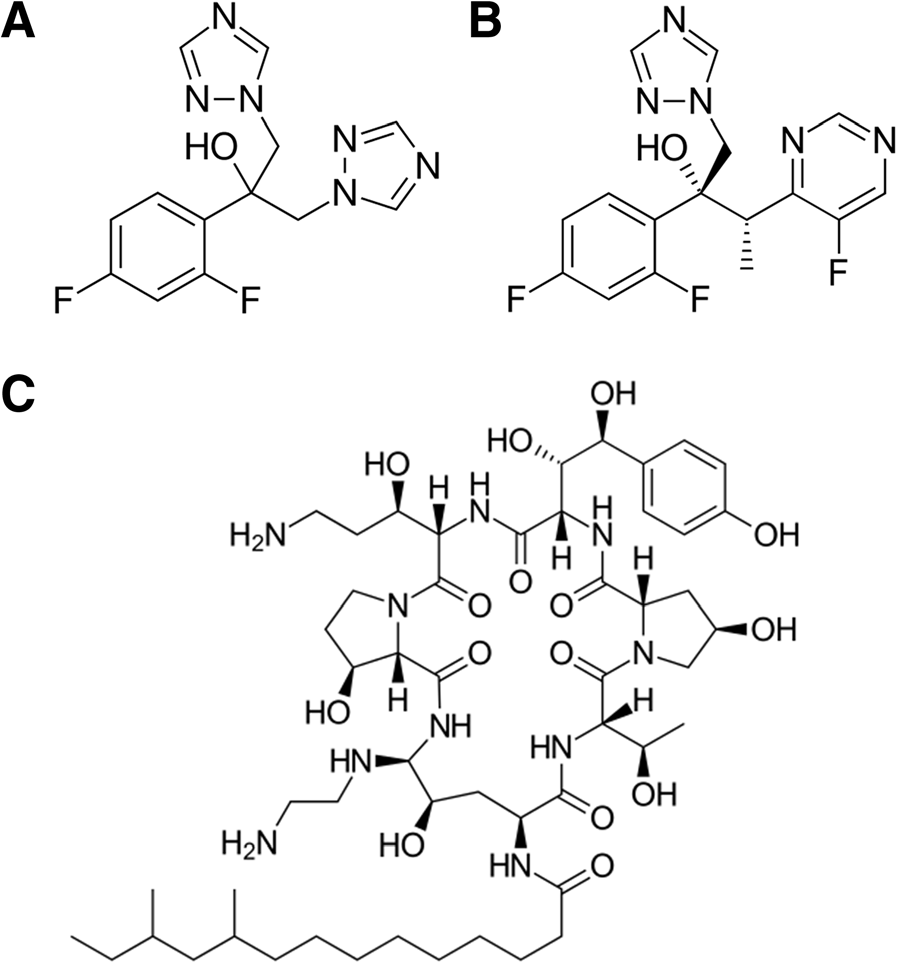
Structural formula of fluconazole (**a**), voriconazole (**b**) and caspofungin (**c**)

In the present study, the interactions between pistachio hull EO and the selected antifungal compounds were antagonistic (FIC index > 4) and droplet size distribution was used to investigate the mechanisms responsible for such interactions. PdI values close to 1.0 are indicative of polydispersed systems [29], whereas values close to 0.6 suggesting monomodal systems. Interestingly, amongst the antifungal used, caspofungin is the most hydrophilic one as shown by its logP value of − 4.59. This property is due to the numerous hydroxyl groups, which makes it able to donate and accept 16 and 18 hydrogen bonds respectively (Fig. 2c). This allows an excellent dispersion of the molecule in the aqueous medium leading to a homogeneous droplet system. In fact, addition of pistachio hull EO to the system did not lead to substantial changes in the average diameter of the droplets or in the PdI of the culture medium. On the contrary, addition of the EO to the system containing either fluconazole or voriconazole (Fig. 2a and b) led to a system stabilization as highlighted by the decrease both of the droplet diameter average then of the PdI. These two drugs, which only differ for the presence of one more fluorine atom on position 5 of the pyrimidine ring and a 2-butanol as aliphatic chain instead of 2-propanol in the voriconazole (Fig. 2b), are less hydrophilic and possess a smaller steric encumbrance compared to caspofungin (Table 4). In fact, fluconazole and voriconazole are able to donate a single hydrogen bond and to accept 7 and 8 hydrogen bonds, respectively. Therefore, on the contrary of which happens in presence of caspofungin, fluconazole and voriconazole are not able to stabilize alone the system but at the same time, the addition of EO, which behaves like a non-ionic surfactant, stabilise the emulsions by reducing interfacial tension and promote steric repulsion between droplets [30]. The main mechanism for emulsion destabilization is actually associated with Ostwald ripening, which induces formation of larger droplets [31]. PdI values range from 1.0 to 0.688 and 0.426 for EO + fluconazole and EO + voriconazole, respectively, reflecting the homogeneity in the droplet size distribution of the new stabilized system. These events could be responsible for the difficulty of both EO and drugs to carry out their antifungal activity against *Candida* strains, which results in an antagonistic effect.

However, considering the interactions between EOs and antimicrobials, it is important to take into account the complex composition of EOs, which makes it rather difficult to predict mode of interaction, especially since the pharmacokinetic profiles are not elucidated. Therefore, the use of individual bioactives could be more commercially viable, with easier standardization in terms of activity, mechanisms of action, pharmacodynamics and pharmacokinetics.

## Conclusions

In summary, the results of the present study showed that bioactives present in pistachio hulls EO are effective against a range of clinical strains of *Candida*, some of whom resistant to antifungal compounds. Further studies need to be performed to elucidate the mechanisms responsible for the activity and the interactions with antifungal compounds.

## Abbreviations

EO: Essential oil
MFC: Minimum Fungicidal Concentration
MIC: Minimum Inhibitory Concentration
